# Neurocognitive impact of Zika virus infection in adult rhesus macaques

**DOI:** 10.1186/s12974-022-02402-4

**Published:** 2022-02-07

**Authors:** Denise C. Hsu, Kesara Chumpolkulwong, Michael J. Corley, Taweewun Hunsawong, Dutsadee Inthawong, Alexandra Schuetz, Rawiwan Imerbsin, Decha Silsorn, Panupat Nadee, Jumpol Sopanaporn, Yuwadee Phuang-Ngern, Chonticha Klungthong, Matthew Reed, Stefan Fernandez, Lishomwa C. Ndhlovu, Robert Paul, Luis Lugo-Roman, Nelson L. Michael, Kayvon Modjarrad, Sandhya Vasan

**Affiliations:** 1 US Military HIV Research Program, Walter Reed Army Institute of Research, Silver Spring, MD 20910 USA; 2 Armed Forces Research Institute of Medical Sciences, Bangkok, 10400 Thailand; 3 Henry M. Jackson Foundation for the Advancement of Military Medicine, Inc, Bethesda, MD 20817 USA; 4 Division of Infectious Diseases, Department of Medicine, Weill Cornell Medicine, New York, USA; 5 Feil Family Brain & Mind Research Institute, Weill Cornell Medicine, New York, NY 10021 USA; 6 Missouri Institute of Mental Health, University of Missouri, St. Louis, MO 63143 USA; 7 Center for Infectious Diseases Research, Walter Reed Army Institute of Research, Silver Spring, MD 20910 USA; 8 Emerging Infectious Disease Branch, Walter Reed Army Institute of Research, Silver Spring, MD 20910 USA

**Keywords:** Zika virus, Neurocognition, Neurobehavior, Neuro-inflammation

## Abstract

**Background:**

Zika virus (ZIKV) is a mosquito-transmitted flavivirus that affects many regions of the world. Infection, in utero, causes microcephaly and later developmental and neurologic impairments. The impact of ZIKV infection on neurocognition in adults has not been well described. The objective of the study was to assess the neurocognitive impact of ZIKV infection in adult rhesus macaques.

**Methods:**

Neurocognitive assessments were performed using the Cambridge Neuropsychological Test Automated Battery (CANTAB) via a touch screen and modified Brinkman Board before and after subcutaneous ZIKV inoculation. Immune activation markers were measured in the blood and cerebral spinal fluid (CSF) by multiplex assay and flow cytometry.

**Results:**

All animals (*N* = 8) had detectable ZIKV RNA in plasma at day 1 post-inoculation (PI) that peaked at day 2 PI (median 5.9, IQR 5.6–6.2 log_10_ genome equivalents/mL). In all eight animals, ZIKV RNA became undetectable in plasma by day 14 PI, but persisted in lymphoid tissues. ZIKV RNA was not detected in the CSF supernatant at days 4, 8, 14 and 28 PI but was detected in the brain of 2 animals at days 8 and 28 PI. Elevations in markers of immune activation in the blood and CSF were accompanied by a reduction in accuracy and reaction speed on the CANTAB in the majority of animals.

**Conclusions:**

The co-occurrence of systemic and CSF immune perturbations and neurocognitive impairment establishes this model as useful for studying the impact of neuroinflammation on neurobehavior in rhesus macaques, as it pertains to ZIKV infection and potentially other pathogens.

**Supplementary Information:**

The online version contains supplementary material available at 10.1186/s12974-022-02402-4.

## Introduction

Zika virus (ZIKV) is a mosquito-borne pathogen of the *Flaviviridae* family of single-stranded positive RNA viruses, which includes Dengue virus (DENV), Yellow fever virus (YFV), Japanese encephalitis virus (JEV), West Nile virus (WNV) and tick-borne encephalitis virus (TBEV) [1]. At the height of the 2016 public health emergency, over 8000 cases of ZIKV infection were reported per week in Brazil. By that point, over 8604 babies had been born with a constellation of congenital malformations that eventually became known as congenital Zika syndrome [2, 3].

The outbreak in the Western hemisphere revealed that ZIKV is highly neurotropic, as it was repeatedly isolated from brain tissue [4–6]. ZIKV has also been demonstrated to infect human neural progenitor cells, attenuating their growth and accelerating cell death [7, 8]. In utero infection is associated with microcephaly and other congenital malformations [9–11]. Furthermore, infants seemingly asymptomatic at birth may eventually develop abnormalities detected by brain imaging or in subsequent neurodevelopmental evaluations [12–15]. In adults, ZIKV infection is generally asymptomatic, manifesting in 20% as a syndrome of maculo-papular rash, fever, conjunctivitis, arthralgia, myalgia and headache [16–19]. Guillain–Barré syndrome [11, 20, 21] and encephalitis [22] have also been reported. The differential impact of ZIKV on the CNS in fetal vs adult infection is likely because undifferentiated neurons during the early stages of neurogenesis in fetal brains, are highly permissive to ZIKV infection, whereas differentiated neurons, representative of adult brain neurons, are relatively resistant to the virus [23]. In one case study of a ZIKV-infected adolescent, neurocognitive changes (including slow processing speed, impaired visuospatial learning and memory) persisted for more than 4 months post-infection [24]. Otherwise, the impact of ZIKV infection on adult neurocognition has not been well described.

Nonhuman primate (NHP) models are useful in the study of viral neuropathogenesis as they are closely related to humans, especially with respect to brain pathology, which is typically difficult to investigate in humans. Furthermore, NHPs are highly permissive to infection by ZIKV and pathogenesis mimics that in humans [25]. Studies of ZIKV in NHPs were first reported in 1952 by Dick et al., who noted the neurotropism of ZIKV in mice and demonstrated intracerebral inoculation of monkeys resulting in viremia [26]. Subsequent studies of ZIKV neuropathogenesis in NHPs have mostly focused on in utero infection [27, 28]. Loss of brain volume and neuronal progenitor cells, gliosis and hippocampal injuries have been shown [27–29]. A study of postnatal infection at 5 weeks after birth found ZIKV RNA and inflammatory infiltrates in the central nervous system. Structural abnormalities were also evident on magnetic resonance imaging at 3–6 months post-infection. Furthermore, ZIKV-infected animals displayed reduced hostility and anxiety behaviors than controls during a stress-induction paradigm [30].

There remains a knowledge gap in the impact of ZIKV infection on adult neurocognition. In this study, we assessed the impact of ZIKV on neurocognition in adult infection in rhesus macaques using the Cambridge Neuropsychological Test Automated Battery (CANTAB, [31, 32]) system for NHPs and a fine motor speed and dexterity task modeled from the Brinkman Task [33]. The CANTAB system for NHPs contains a series of computerized tests of memory, attention and executive function that is administered through a touch-sensitive screen linked to a food reward dispenser. It has been developed and validated for the assessment of neurocognitive deficits in both animals and humans [34, 35]. We sought to correlate immune and neurobehavioral findings to model the impact of ZIKV infection on CNS function in adult rhesus macaques.

## Methods

### Animal selection

Animals were housed at the AAALAC International-accredited, Armed Forces Research Institute of Medical Sciences (AFRIMS) in Bangkok, Thailand. The protocol was approved by the AFRIMS Institutional Animal Care and Use Committee. Research was conducted in compliance with Thai laws, the Animal Welfare Act and other U.S. federal statutes and regulations relating to animals and experiments involving animals and adheres to principles stated in the Guide for the Care and Use of Laboratory Animals, 2011 edition [36].

### Study design

**Fig. 1:**
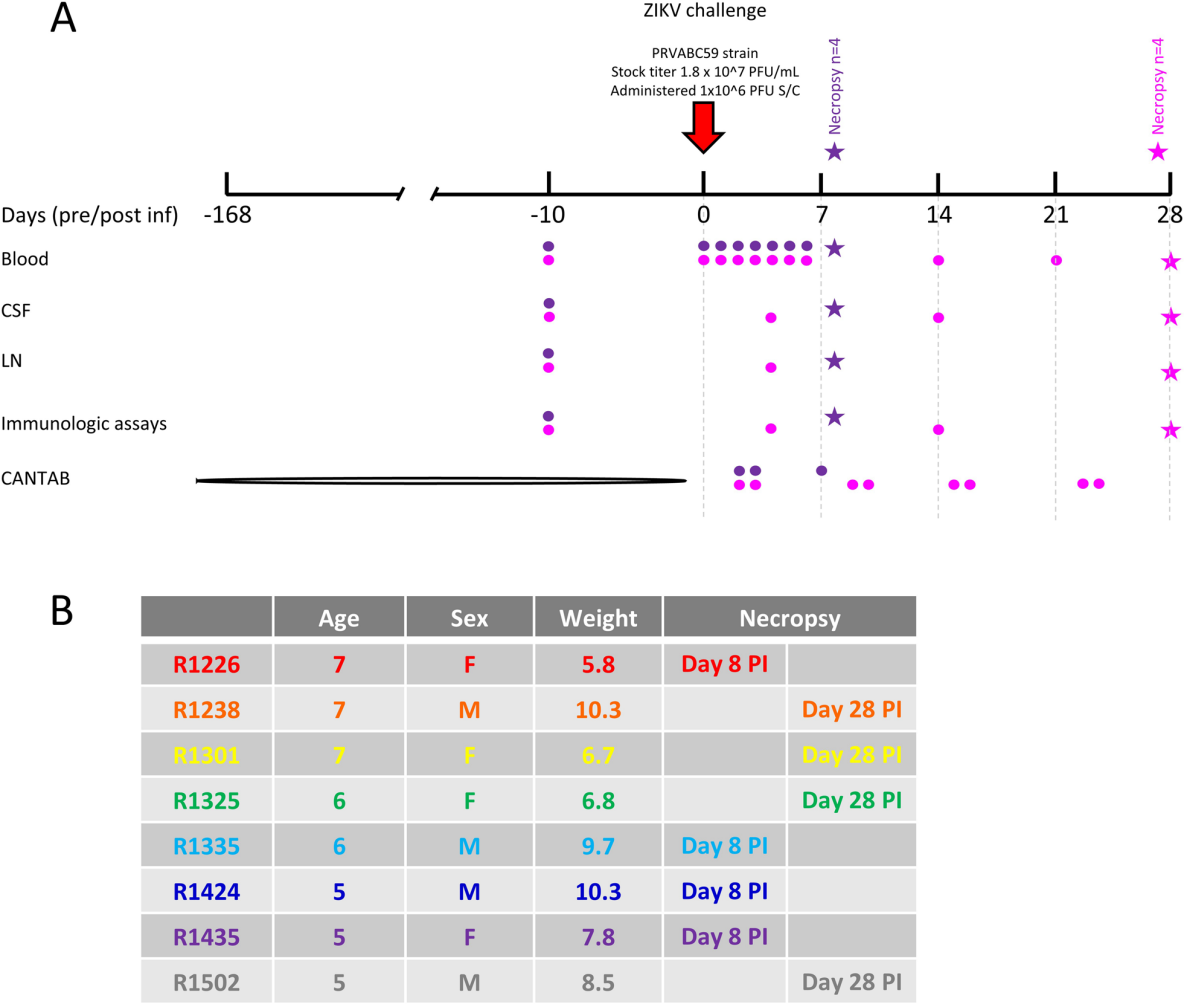
Study schema. Eight adult, Indian-origin rhesus macaques were trained to undergo CANTAB assessments for 24 weeks. Animals were inoculated with ZIKV at day 0. Animals were then divided into 2 groups and were euthanized early (*n* = 4, day 8 PI, purple) or late (*n* = 4, day 28 PI, pink). Blood, CSF and lymph node sampling and CANTAB assessments are indicated for animals in each group (**A**). Characteristics of the animals including age, sex, weight and group assignment are listed (**B**)

Eight adult, Indian-origin rhesus macaques (*Macaca mulatta*, 4 males, 4 females), median age 6 (range 5–7) years, median weight 8.2 (range 5.8–10.3) kg were included in the study. Prior to entry into the study, animals were screened to confirm normal physical condition, complete blood count, blood chemistry and sero-negativity to mosquito-borne Flaviviruses endemic to Thailand, including DENV (serotypes 1, 2, 3 and 4), JEV and ZIKV. Animals were trained to undergo neurocognitive assessment for 24 weeks prior to ZIKV inoculation at day 0. Animals were then divided into two groups and were humanely euthanized early (day 8 PI, *n* = 4) or late (day 28 PI, *n* = 4). In both groups, plasma ZIKV RNA was monitored daily for 6 days PI. Animals in the late group also underwent CSF collection at days 4, 14 and during necropsy and lymph node collection at day 4 and during necropsy (Fig. 1A). Characteristics of the animals including age, sex, weight and group assignment are listed in Fig. 1B.

### NHP CANTAB training and assessment

The Monkey CANTAB Intellistation with Pellet Reward (Lafayette Instrument, Lafayette, IN, USA) was used to perform neurocognitive testing. In this study, rhesus macaques were assessed using the Motor Screening Task (MOT) and Self-Ordered Spatial Search (SOSS). The MOT assesses sensorimotor deficits and reaction speed. The SOSS assesses frontal lobe working memory functions. Both of these tests have been widely used in humans and monkeys, across a range of ages [35, 37–40] and pathological conditions, including neurodegenerative [41, 42], neuropsychiatric [43], infectious [44, 45] and the effects of psychotropic agents [46–49].

The animals were trained to undergo CANTAB assessments 2–3 times a week, in 5–25 min afternoon sessions, for 24 weeks (Additional file 1: Fig. S1). Step 1: To familiarize the animal, CANTAB Intellistation was brought in front of the animal’s cage and the animal was allowed to explore it and take rewards from the dispenser in response to an auditory stimulus from a manual clicker. Step 2: A visual stimulus, filling the whole screen was presented and the animal was rewarded for touching the screen. Step 3: The visual stimulus reduced in size iteratively following 10 consecutive correct responses. The animal was rewarded after each correct response. Step 4 (Motor Screening Task, MOT): A colored stimulus of fixed-size appeared on the screen in random locations and the animal received a reward after touching the stimulus. Animals were given 10 min to complete as many motor tasks as possible. Accuracy on the motor task was calculated as the percentage of correct responses divided by the total responses; and speed was determined by the number of tasks the animal was able to complete within 10 min. Step 5 (Self-Ordered Spatial Search, SOSS): A set of visual stimuli were presented together at the start of each individual trial. The animal was required to touch each stimulus without returning to a stimulus already selected. Trials were defined as completed when the animal either touched all of the targets without repetition (correct), touched a target that had previously been selected in that trial (error), or failed to touch a target within 30 s (omission). After an inter-trial interval of 5 or more seconds, a new trial was presented with stimuli randomly assigned to a new location on the screen. A blank screen was presented briefly after each response. Animals were required to complete 40 SOSS tasks. Accuracy on SOSS was calculated as the percentage of correct responses divided by the total responses; and speed was determined by the time taken to complete 40 tasks.

Sex differences were observed in the rate of learning between male and female animals for SOSS training and, thus, required a shift in the presentation of tasks and rewards to motivate the female animals.

### Modified Brinkman Board

This task assessed fine motor speed and dexterity. It involved the retrieval of raisins from a board that contained wells oriented in different directions (i.e., vertically, horizontally and diagonally) with different depths and angles [33, 50, 51]. The task was administered using two boards that differed in task complexity vis-à-vis increasing the depth of wells, altering the angle of the wells and increasing the number of wells (from 6 wells on the first board to 9 wells on the second board). Animals were assessed once pre-infection and twice per week post-infection, on the same day as the CANTAB assessment. The dependent variables included the number of raisins retrieved and the time taken to clear the board of raisins.

### Assays to screen for DENV, JE and ZIKV sero-positivity

Hemagglutination inhibition (HAI) assays were used to screen for flavivirus sero-positivity by measuring of DENV1-4, JEV and ZIKV antibodies in serum collected 2 weeks prior to study entry. Serum was heat inactivated at 56 °C for 30 min and non-specific protein binding was reduced by acetone extraction. Two-fold serially diluted serum samples were added to individual sucrose–acetone extracted viral antigens. Hemagglutination was detected by the addition of goose red blood cells. HAI titer was defined as the highest dilution of serum that inhibited hemagglutination. Only NHPs with HAI titer < 10 for all viral antigens were included in this study.

### Verification of infectious ZIKV by plaque assay

Infectious ZIKV was quantified by plaque assay using LLC-MK2 cell. Viral supernatant was ten-fold serial diluted, starting from 1:10 to 1:10,000, with tissue culture media, Minimum Essential Media (MEM) supplemented with 10% heat inactivated FBS (Invitrogen, USA). Then, 100 µL of diluted sample was added onto triplicate wells of LLC-MK2 cell monolayer in 12-well plates. After incubation for 1 h at room temperature (RT) on a rocker platform, the excess amount of inoculum was removed. First overlay medium with 1.8% LMP was added and the agar was allowed to solidify at RT prior to incubation at 35 °C, 5% CO_2_ for 4 days. Virus-infected cells or plaque forming units (PFU) were visualized after staining with second overlay medium containing 4% Neutral red (Sigma, USA) on the following day. The average number of plaque from triplicate wells in each dilution was used to calculate ZIKV titer per milliliter (PFU/mL).

### Zika virus challenge

ZIKV PRVABC59 strain (stock titer of 1.8 × 10^7^ PFU/mL, provided by R. De La Barrera, WRAIR) was used [52]. Animals were inoculated with 1 × 10^6^ PFU subcutaneously in the left leg at week 0. Animals were observed three times per day for clinical signs of disease including inappetence, dehydration and lethargy. Intra-rectal temperature was measured daily using pole collar chair restraint for 10 days after ZIKV inoculation.

### Quantitation of ZIKV RNA

Plasma, CSF and tissue ZIKV-RNA levels were measured using real-time quantitative PCR [53, 54]. Viral RNA was extracted from 140 µL plasma and CSF using the QIAamp viral RNA mini kit (QIAGEN, Germany). For tissue samples, RNeasy Mini Kit (QIAGEN, Germany) was used as per the manufacturer’s instruction. ZIKV real-time quantitative RT-PCR was performed using a method modified from Lanciotti et al. [55]. Two primer/probe sets were used: (1) ZIKV 1086 forward, ZIKV 1162c reverse primers and ZIKV 1107-FAM probe [56]; and (2) ZIKV 4434 forward, ZIKV 4524c reverse primes and ZIKV 4479c-FAM probe [57]. In each experiment, 3 internal controls (no template control, NTC, negative extraction control, NEC and positive extraction control, PCE) and 6 in vitro transcribed RNA standards (at 5, 50, 5 × 10^2^, 5 × 10^3^, 5 × 10^4^, 5 × 10^5^ copies/reaction) were included. All assays were performed using the SuperScript III Platinum One-Step Quantitative RT-PCR kit (Invitrogen), as per manufacturer’s instructions, using the Applied Biosystems 7500 Fast Real-Time PCR systems (Life Technologies). Limit of quantification of the assay was 5 copies. The amount of ZIKV in GE/mL in controls and samples were calculated using the formula GE/ml = (copies per reaction x eluted RNA vol × 1000 ul)/ (RNA vol used in the reaction (ul) x serum vol used (ul)).

### Measurement of plasma and CSF cytokine levels

Plasma and CSF soluble markers of immune activation (including G-CSF, GM-CSF, IFNγ, IL-1β, IL-1ra, IL-2, IL-4, IL-5, IL-6, IL-8, IL-10, IL-12/23(p40), IL-13, IL-15, IL-17A, MCP-1, MIP-1β, MIP-1α, sCD40L, TGF-α, TNF-α, VEGF, and IL-18) were quantified using the MILLIPLEX MAP Non-Human Primate Cytokine Magnetic Bead Panel (EMD Millipore Corporation, Billerica, Massachusetts, USA) as per manufacturer instructions, as previously described [58].

### Flow cytometric assessments of T cell activation

Immunophenotyping was performed on freshly isolated peripheral blood mononuclear cells (PBMC) using Ficoll Separation Medium, Histopaque®-10771 (Sigma-Aldrich, St. Louis, MO, USA), before resuspension in Dulbecco’s Phosphate Buffered Saline (DPBS; Life Technologies, Paisley, UK). Fresh cerebrospinal fluid (CSF) was centrifuged at 400*g* at 4 °C for 10 min. CSF cell pellet was washed with DPBS and used for immunophenotyping.

Cells were stained with Aqua Live/Dead dye (Invitrogen, Eugene, OR, USA) in the presence of 10% mouse IgG (Fc block; Invitrogen, Eugene, OR, USA). Subsequently samples were stained for 20 min at 4 °C with the following antibodies: anti-CD3 PE-CF594 (BD Horizon, San Diego, CA, USA), anti-CD4 Pacific Blue (Biolegend, San Diego, CA, USA), anti-CD8 BV785 (Biolegend, San Diego, CA, USA), anti-CD19 BV605 (Biolegend, San Diego, CA, USA), anti-CD20 BV711 (Biolegend, San Diego, CA, USA), anti-CD16 PE-Cy5 (Biolegend, San Diego, CA, USA), anti-CD56 PE-Cy7 (BD Bioscience, San Jose, CA, USA), anti-CD14 BV650 (BD Horizon, San Diego, CA, USA), anti-CD38 PE (Caprico Biotechnologies, Norcross, GA, USA), and anti-HLA-DR APC-H7 (BD Pharmingen, San Diego, CA, USA). They were then permeabilized with 1X BD Perm/Wash buffer (BD Bioscience, San Jose, CA, USA) for 15 min at 4 °C before staining with anti-Ki67 AlexaFluor488 (BD Pharmingen, San Diego, CA, USA) for 30 min at 4 °C. After staining cells were resuspended in 1% formaldehyde and acquired immediately using a custom-built LSRFortessa flow cytometer (BD, San Jose, CA, USA) and analyzed using FlowJo software version 10.5.3 or higher (TreeStar, Ashland, OR, USA).

### Histopathology

Tissue samples were fixed in 4% paraformaldehyde for 16–24 h, processed in an automatic tissue processor (TP1020; Leica, Buffalo Grove, IL, USA), paraffin embedded, sectioned at 5 um, and mounted onto adhesive microscope slides (BioGnost, Medugorska, Zagreb, Croatia).

Slides were stained with Harris’s hematoxylin solution (In-House Prep.) for 10 min, washed and stained with Eosin solution (In-House Prep.) for 45 s, dehydrated with ethanol, and cleared with xylene prior to mounting with toluene-based mounting medium (Thermo Fisher Scientific, Kalamazoo, MI, USA).

Slides from basal ganglia and parietal areas of the brain were reviewed by a board-certified veterinary pathologist, in comparison to similar tissue sections from control animals.

### Statistical analyses

Analysis of virologic and immunologic data was performed using GraphPad Prism v8.4.3 (GraphPad Software). Comparisons between different time-points in the same animals were performed using Wilcoxon matched-pairs signed rank test. Pre-infection CANTAB performance was established by generating mean and standard deviation (SD) using the last 4 sessions of assessment prior to ZIKV inoculation (Additional file 2: Table S1). To quantify changes post-infection, individualized Z-scores (number of SD below or above the pre-infection mean) were generated using data from each animal post-ZIKV inoculation. This method of quantification of neurocognitive impairment was based on the work of Weed et al. [44] and is advantageous as it takes into account individual variability in performance in each animal pre- and post-infection. Impairment was defined as > 2.5 SD (corresponded to 99% confidence interval around the baseline mean) reduction in accuracy for both MOT and SOSS, > 2.5 SD reduction in the number of MOT performed within 10 min or > 2.5 SD increase in time to complete 40 SOSS tasks.

## Results

### Clinical evaluations of ZIKV-inoculated rhesus macaques

Eight rhesus macaques were infected with ZIKV through subcutaneous ZIKV inoculation. Apart from transient reduction in appetite, animals did not exhibit any signs of clinical illness including fever (intra-rectal temperature > 103°F).

### ZIKV RNA was detectable in plasma and lymph node tissues in all animals post-infection

**Fig. 2:**
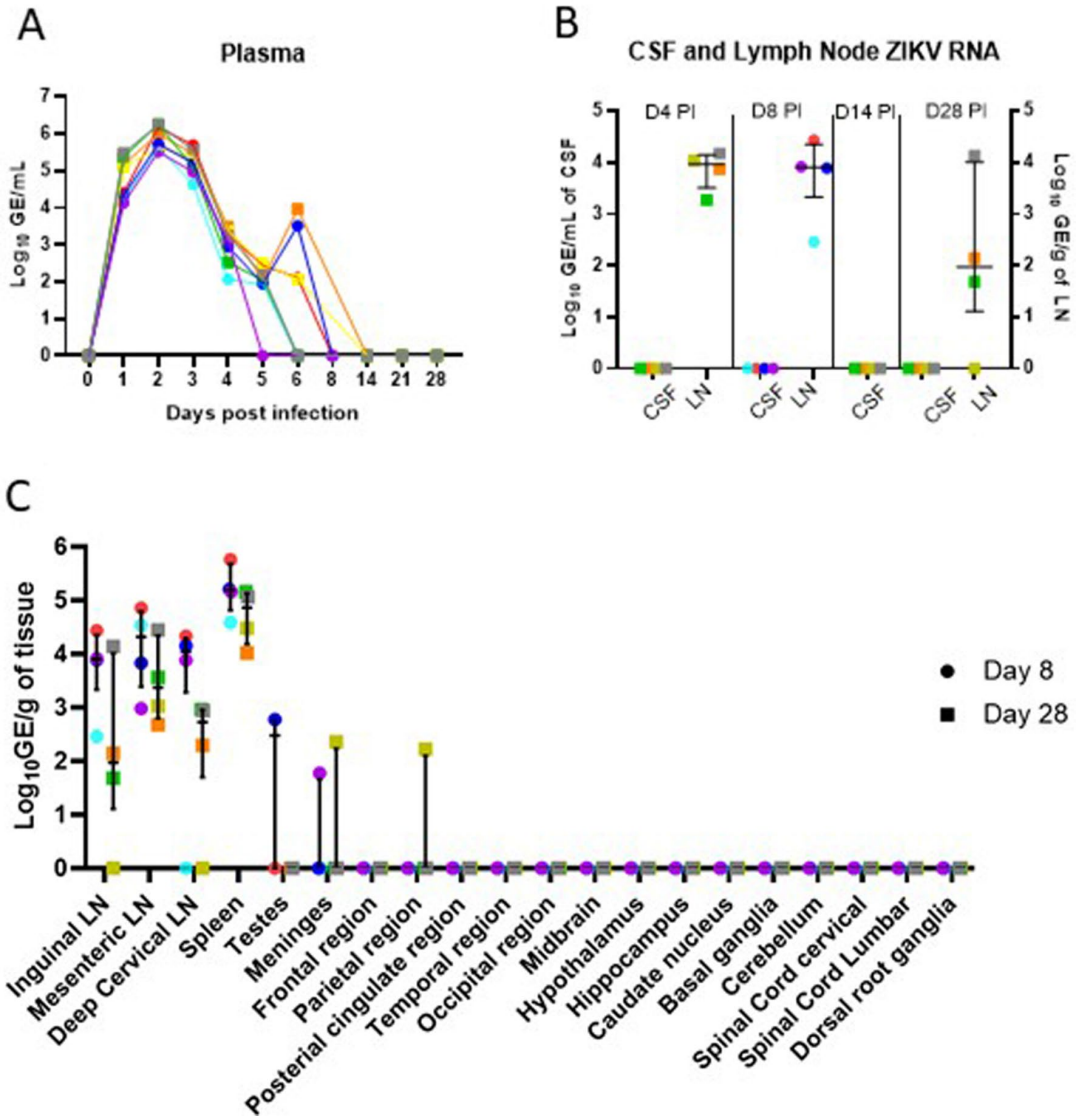
ZIKV RNA levels. All animals had detectable ZIKV RNA in plasma at day 1 post-inoculation (PI) that peaked at day 2 PI and became undetectable in plasma by day 14 in all animals (**A**). ZIKV RNA was not detected in the CSF in the time points sampled (**B**). ZIKV RNA was detected in the central nervous system in 2 animals and in lymph node tissues in all animals at the time of necropsy (**C**). Each color represents data from individual animals as listed in Fig. 1B, animals in the early group are represented by circles and the late group by squares

All animals had detectable ZIKV RNA in their plasma at day 1 PI that peaked at day 2 PI (median 7.55 × 10^5^, IQR 4.1 × 10^5^–1.69 × 10^6^, GE/mL). Plasma ZIKV RNA declined rapidly and became undetectable in plasma by day 8 PI in all animals in the Early group and day 14 in all animals in the Late group (Fig. 2A). ZIKV RNA was detectable in lymph node tissues in 4 of 4 animals at day 4 (Late group) and at day 8 (Early group) and persisted in 3 of 4 animals (Late group) at day 28 PI (Fig. 2B). ZIKV RNA was not detected in CSF supernatant at any of the time points measured (day 8 in *n* = 4 Early group, days 4, 14 and 28 in *n* = 4, Late group, Fig. 2B). Of the 15 different areas of the nervous system assessed (Fig. 2C), only low levels of ZIKV RNA were detected in the meninges (R1301 at 228 GE/mg at day 28 PI and R1435 at 60 GE/mg at day 8 PI) and the parietal region (R1301 at 166 GE/mg at day 28 PI).

### Transient immune activation in the peripheral blood and CSF

**Fig. 3:**
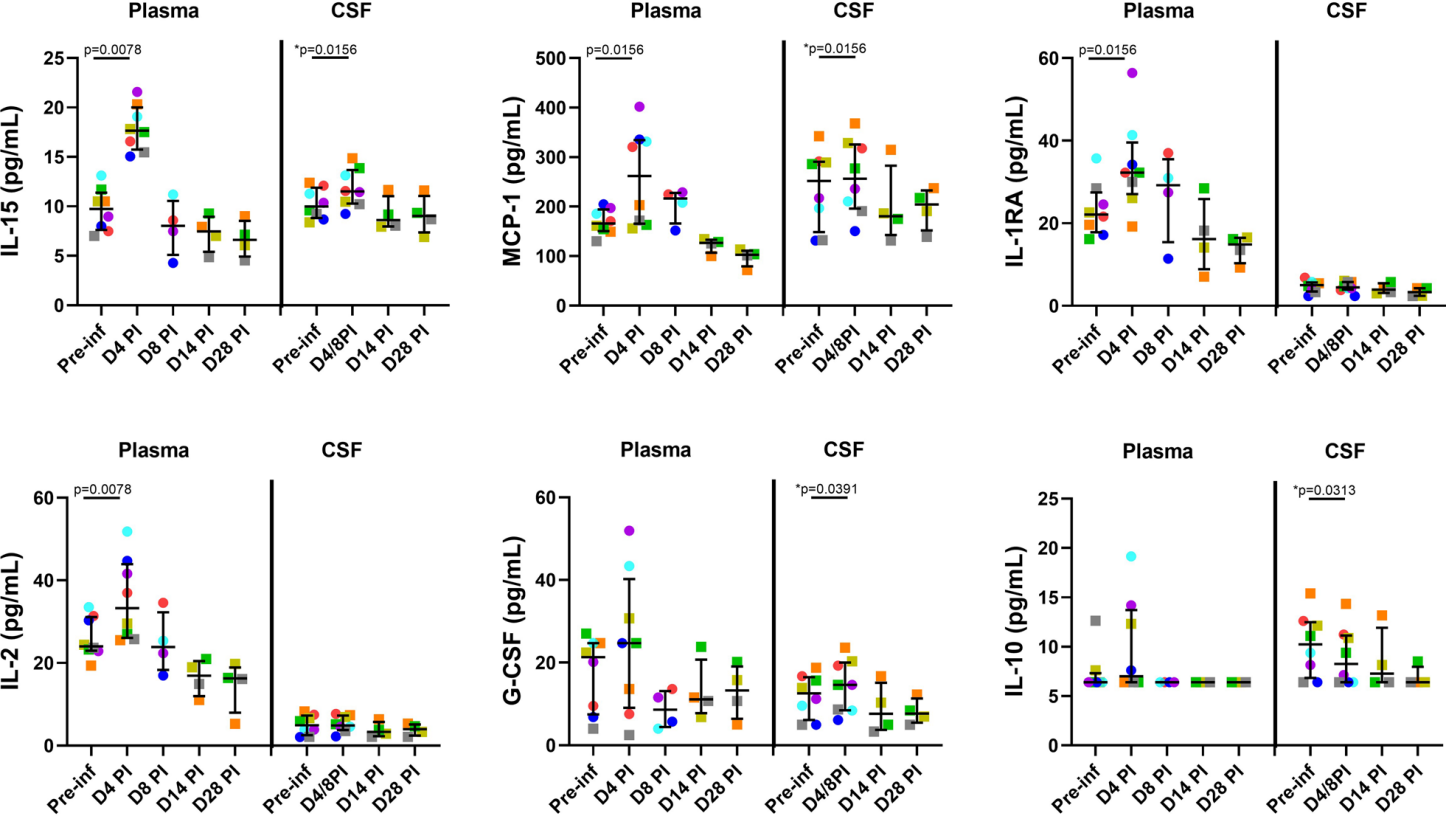
Plasma and CSF soluble markers of immune activation. Transient increases in soluble markers of immune activation were observed in the plasma (interleukin, IL-15, monocyte chemoattractant protein-1, MCP-1, IL-1 receptor antagonist, IL-1RA and IL-2) and CSF (IL-15, MCP-1, granulocyte colony stimulating factor, G-CSF) early post-ZIKV infection. Each color represents data from individual animals as listed in Fig. 1B. *P values for CSF comparisons were based on early post-inoculation (PI) results from all 8 animals (Late group, *n* = 4 at day 4 PI, squares and Early group, *n* = 4 at day 8 PI, circles)

**Fig. 4:**
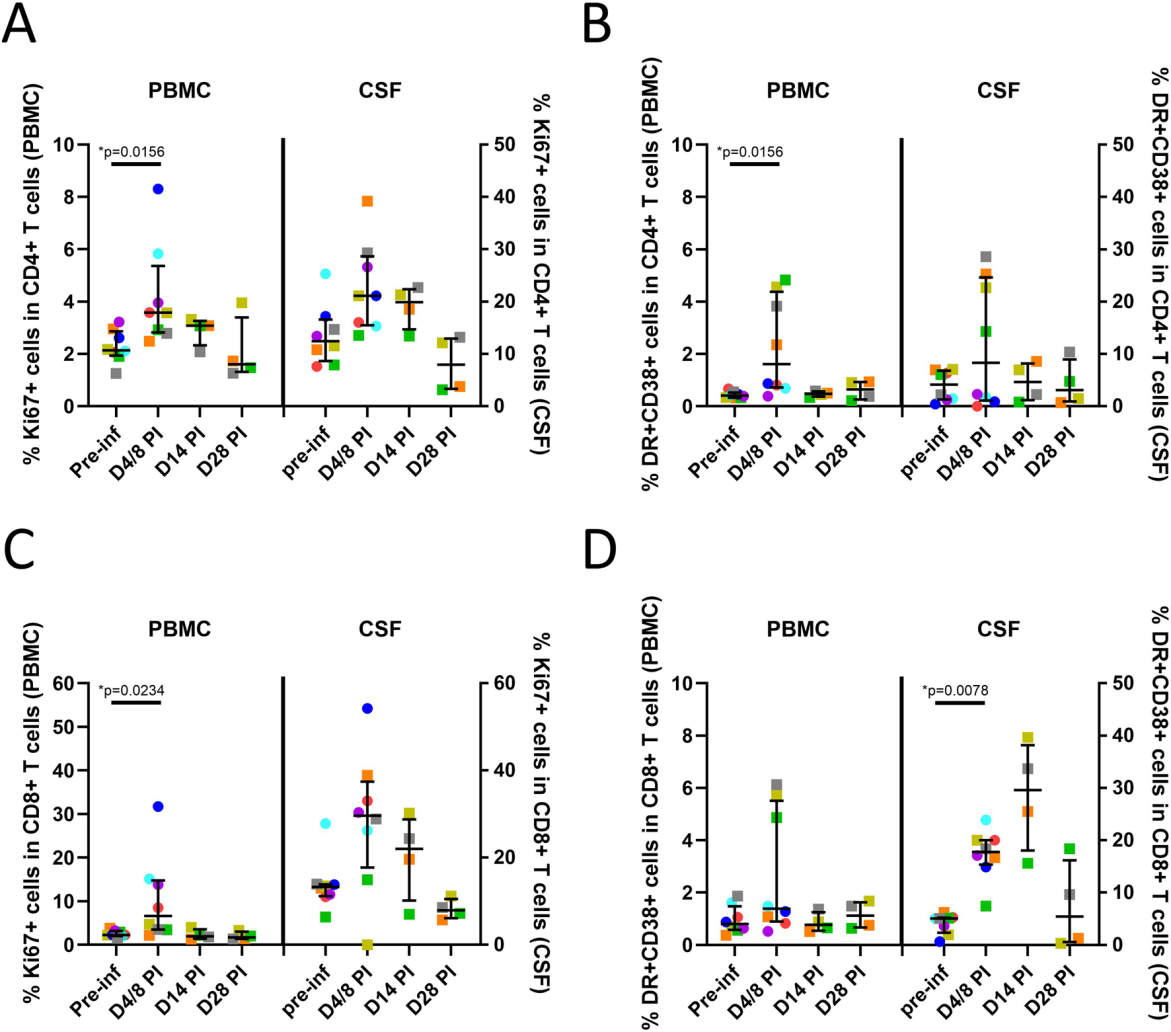
Cellular markers of immune activation. Increases in cellular markers of immune activation were identified in CD4 and CD8 T cells in the peripheral blood and in CD8 T cells in the CSF. Percentages of Ki67 + cells (**A**) and HLA-DR + CD38 + (**B**) in CD4 T cells and percentages of Ki67 + cells (**C**) and HLA-DR + CD38 + (**D**) in CD8 T cells are shown. Each color represents data from individual animals as listed in Fig. 1B. *P values were based on early post-inoculation (PI) results from all 8 animals (Late group, *n* = 4 at day 4 PI, squares and Early group, *n* = 4 at day 8 PI, circles)

Significant increases in a number of soluble markers of immune activation including IL-15 (*p* = 0.0078), MCP-1 (*p* = 0.0156), IL-1 RA (*p* = 0.0156), and IL-2 (*p* = 0.0078) were observed in plasma at day 4 PI (Fig. 3), that then reduced and normalized by day 14 PI. Similar trends were observed in the CSF and were statistically significant for IL-15 (*p* = 0.0156), MCP-1 (*p* = 0.0156) and G-CSF (*p* = 0.0391) when results from animals in the Early (*n* = 4, day 8 PI) and Late (*n* = 4, day 4 PI) groups were combined (Fig. 3). Levels normalized by day 14 PI. In addition, a pattern of reduction in CSF IL-10 levels was also seen post-infection (Fig. 3). When compared to pre-infection, significant increases in cellular markers of immune activation were also identified in CD4 (Ki67 + , *p* = 0.0156 and CD38 + HLA-DR + , *p* = 0.0156, Fig. 4A, B) and CD8 (Ki67 + , *p* = 0.0234, Fig. 4C) T cells in the peripheral blood and in CD8 (CD38 + HLA-DR + , *p* = 0.0078) T cells in the CSF (Fig. 4D), when data from animals in the Early (*n* = 4, day 8 PI) and Late (*n* = 4, day 4 PI) groups were combined. Interestingly, CD4 and CD8 T cell activation mostly resolved in the peripheral blood by day 14 PI but CSF CD38 + HLA-DR + CD8 T cell frequency peaked at day 14 PI and remained elevated in 2 of 4 animals (when compared to each animals’ pre-infection level) at day 28 PI. Thus, ZIKV infection was associated with early immune activation in both the peripheral blood and the CSF, but largely normalized by day 28 PI, with the exception of CSF CD8 T cell activation.

Hematoxylin and eosin-stained slides from the basal ganglia and parietal areas of each animal were reviewed by a board-certified veterinary pathologist. These sections were chosen as they represent superficial and deep parts of the brain. No pathological findings were observed.

### Neurocognitive impairment post-ZIKV infection

Pre-infection CANTAB performance for each animal was established as the mean of the scores from the last 4 sessions of assessment prior to ZIKV inoculation (Additional file 2: Table S1). Median (IQR) of the pre-infection scores for all 8 animals were 80.48% (72.01–88.13) for MOT accuracy, 69.38% (49.53–82.97) for SOSS accuracy, 71.25 tasks (68.25–74.75) for the number of MOT performed within 10 min and 6.54 min (6.33–7.25) for the time required to complete 40 SOSS tasks.

**Fig. 5:**
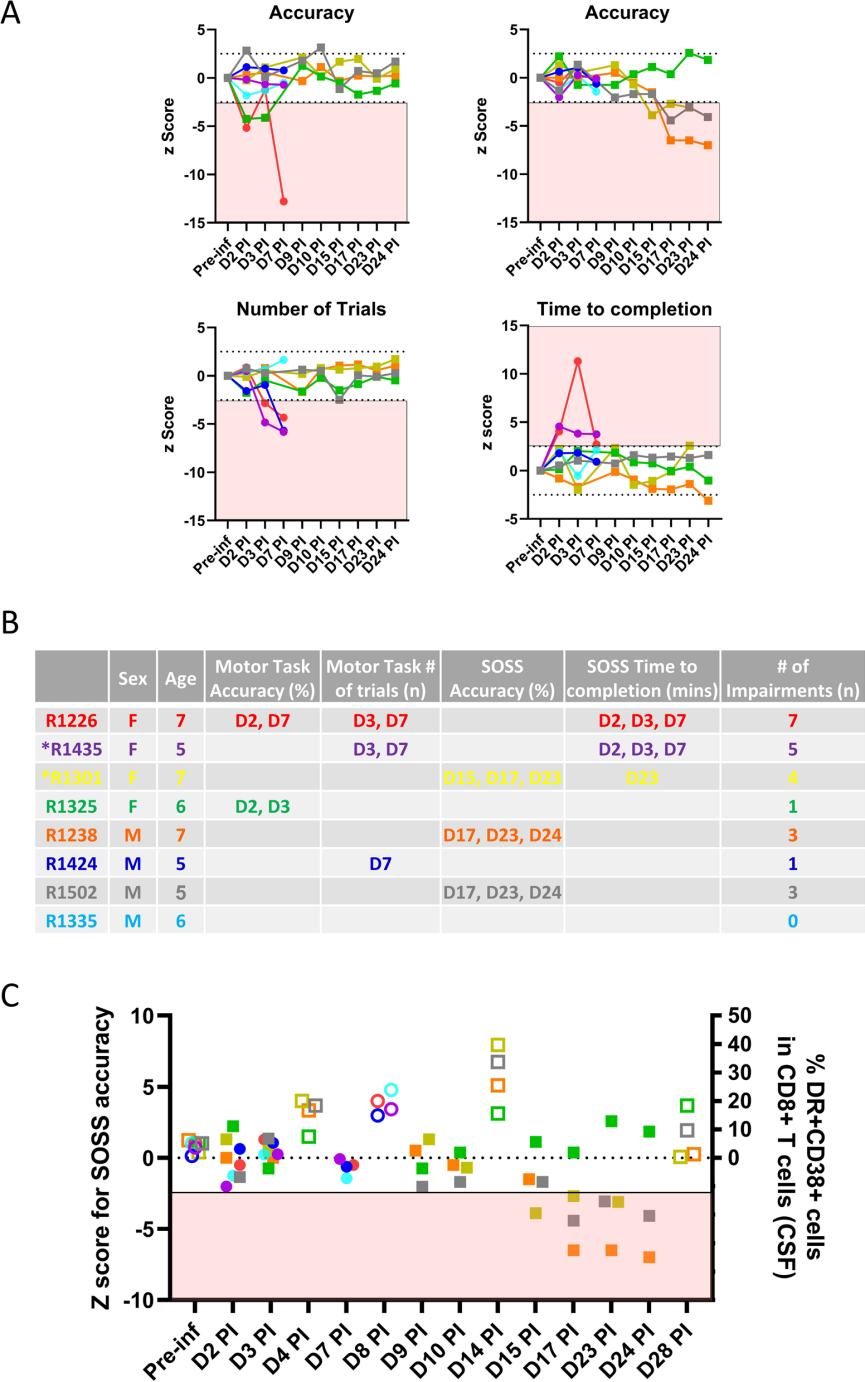
Neurocognitive outcome as assessed by the Cambridge Neuropsychological Test Automated Battery (CANTAB). Individualized Z-scores (number of SD below or above the pre-infection mean) were generated for each animal at each assessment post-ZIKV inoculation (**A**). Impairment (shaded in red) is defined as > 2.5 SD reduction in accuracy for both Motor Screening Task (MOT) and Self-Ordered Spatial Search (SOSS), > 2.5 SD reduction in the number of MOT performed within 10 min or > 2.5 SD increase in time to complete 40 SOSS tasks. Sex, age, timing of occurrence of and the number of impairments recorded are listed for each individual animal (**B**). * Indicates the two animals that had detectable ZIKV RNA in the brain. Impairment on SOSS accuracy (filled symbols) followed the peaking of CSF HLA-DR + CD38 + CD8 T cells (open symbols) in 3 of 4 animals in the Late group (**C**). Each color represents data from individual animals as listed in Fig. 1B, animals in the early group are represented by circles and the late group by squares

To assess changes post-infection, individualized Z-scores (number of SD below or above the pre-infection mean) were generated for each animal at each assessment post-ZIKV inoculation (Fig. 5A). Impairments defined as > 2.5 SD change were identified in 7 of 8 animals (Fig. 5B). Interestingly, female animals displayed more impairments (Fig. 5B); although this may be explained by learning style and performance approach rather than impairment per se. Importantly, impairment in SOSS accuracy followed the peak of CSF CD8 T cell activation at day 14 PI in 3 of 4 animals in the Late group (Fig. 5C), suggesting a potential link between neuroinflammation and neurocognitive impairment. The correlation is not statistically significant and larger experiments would be needed for additional validation of this observation.

**Fig. 6:**
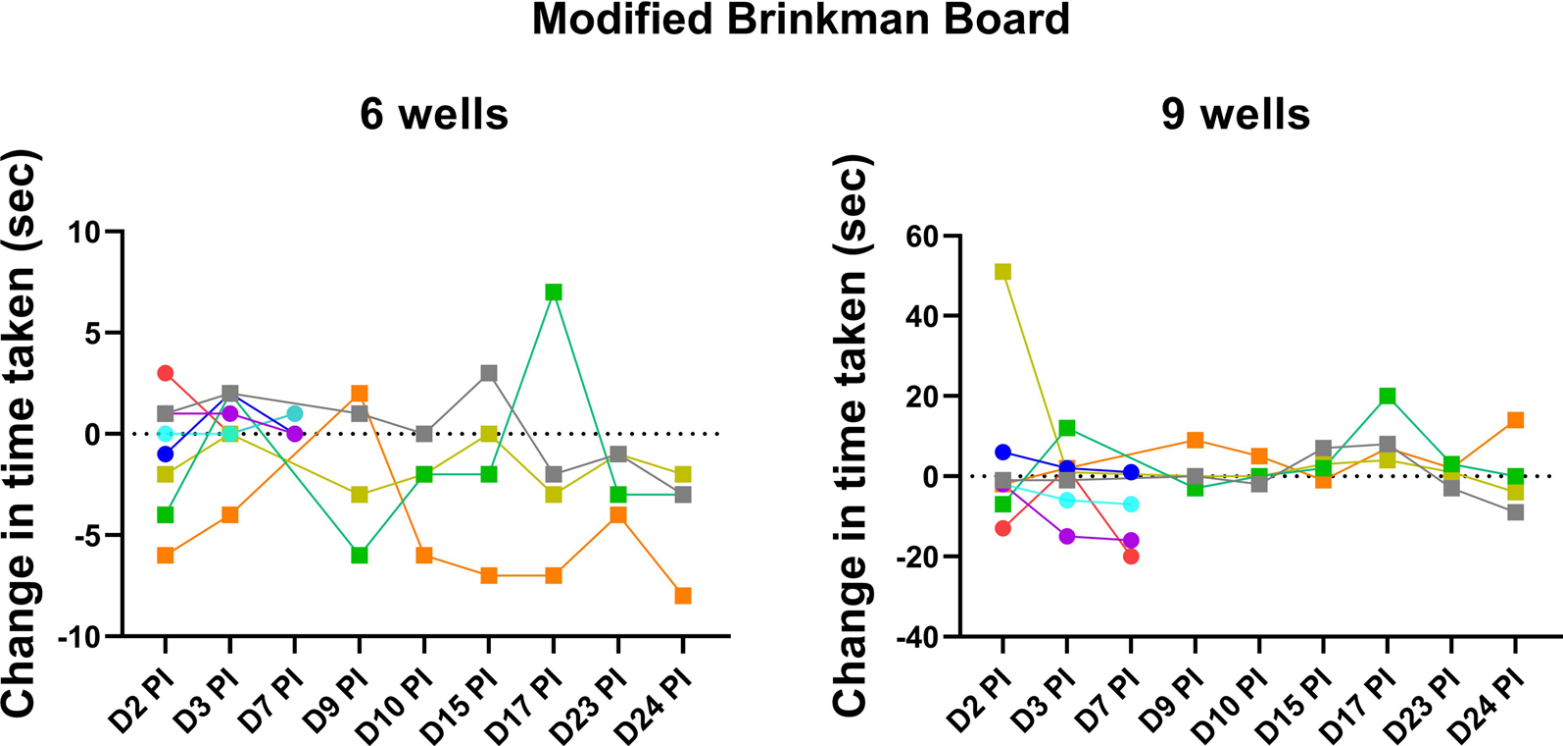
Fine motor assessments on Brinkman Board. All animals retrieved all raisins (6 and 9) from the boards in all assessments. Each color represents data from individual animals as listed in Fig. 1B, animals in the early group are represented by circles and the late group by squares. Animal R1301 (yellow) showed marked increase in time to complete the 9-well board that was not mirrored in the 6-well board. Animal R1325 (green) showed increase in time to complete both 6- and 9-well boards at day 17 post-infection

On the modified Brinkman Board, all animals retrieved all raisins from the two boards in all assessments. No slowing was noted in the majority (6 of 8) of animals (Fig. 6). Animal R1301 at day 2 PI showed a marked increase in time to complete the more complex board with 9 wells, but that was not mirrored in the more simple board with 6 wells in the same test session. Animal R1325 showed an increase in time to complete the simple and complex boards at day 17 PI however, no impairment on CANTAB testing was noted on that day. Thus, these findings suggest that post-infection motor performance was unaffected as assessed by the modified Brinkman Board.

## Discussion

Though not entirely faithful to human disease, NHP models of ZIKV infection have been widely used as they recapitulate many key clinical findings in humans, including rapid control of acute viremia, prolonged viral shedding and fetal pathology after infection in pregnant female animals [28, 59–61]. To our knowledge, this is the first study to utilize adult rhesus macaques to evaluate the neurocognitive impact of ZIKV infection.

Rhesus macaque model of adult ZIKV infection has been well characterized. Animals are infected with ZIKV via subcutaneous inoculation at doses ranging from 10^3^ to 10^6^ PFU [59–64]. ZIKV is detectable in the peripheral blood at day 1 PI, peaks rapidly with plasma viral load of around 5–7 log_10_ RNA copies/mL and then becoming undetectable at 5–10 days PI [59–63]. Sporadic and lower levels of ZIKV is usually observed in urine, genital secretions and saliva. The presence of virus in tissues including lymphoid, CNS and reproductive tissues can be more persistent, lasting weeks to months [60, 63, 64]. Histopathological findings in tissues in adult infection have been variable, ranging from no abnormalities to focal lymphocytic infiltration [64, 65].

Adult ZIKV infection is associated with immune activation with upregulation of cellular markers, including CD69, Ki67 and increases insoluble markers, including MCP-1, IL-2, IL-15, vascular endothelial growth factor (VEGF) and IL-10 in the first few days post-infection [60, 61, 63, 64]. ZIKV-specific binding and neutralizing antibodies emerges at 1 week PI and ZIKV-specific cellular responses soon follows, peaking at around 4 weeks PI [60, 61, 63–65].

Our adult rhesus macaque model of ZIKV infection showed post-infection characteristics that are novel, but also are consistent with the literature. We detected plasma ZIKV RNA at day one after virus inoculation, followed by an early peak and then clearance by 7 to 10 days [59–63] as well as persistence in lymphoid tissue [60, 63, 64]. We were, however, unable to detect ZIKV RNA in the CSF but detected ZIKV RNA in the brains of 2 of 8 animals. Although some studies have documented ZIKV RNA in the CSF in approximately half of the infected rhesus macaques [59–61, 63], the absence of detectable ZIKV RNA in the CSF has also been reported [30, 64, 65]. Our findings support the variability of host responses to ZIKV infection that may be related to viral and/or host risk and protective factors.

In this study, there were transient increases in cellular and soluble markers of immune activation in the peripheral blood, consistent with prior publications [60, 61, 63, 64]. Soluble markers of immune activation normalized in the CSF by day 14 post-infection, however, CD8 T cell activation remained elevated in 2 of 4 animals at day 28 PI. There is a dearth of data describing biomarkers of immune activation in the CSF and, as such, the finding of cellular activation 4 weeks PI is novel. A study with longer follow-up will be required to determine if cellular activation persists and assess its potential impact on long-term neurocognitive performance.

The most unique and significant finding is the identification of neurocognitive impairments (> 2.5 SD change) in 7 of 8 animals utilizing the Monkey CANTAB, which has been adapted from a human neuropsychological testing battery. Impairment in SOSS accuracy followed the peaking of CD8 T cell activation at day 14 PI in 3 of 4 animals in the Late group. Studies in other disease models have shown that both experimentally induced and naturally occurring upper respiratory tract illnesses (URTIs) can influence mood and performance [66, 67]. Furthermore, the effects of influenza on performance can be mimicked by administrations of alpha interferon [68], suggesting that immune activation associated with viral infections can impact cognitive performance. These observations would need to be confirmed in a study with larger number of animals and longer follow-up.

The absence of uninfected controls is a potential limitation of this study. However, the study leveraged the methodological strengths of a longitudinal model in which individual animals served as their own control. This approach yielded statistically significant changes in immune responses in blood and CSF post-infection when compared with samples from the same animal pre-infection. In this study, we were unable to delineate whether the neurocognitive impairment was secondary to the direct effect of ZIKV infection or systemic immune activation. As such, the performance on CANTAB may potentially be confounded by a lack of engagement due to feeling unwell. However, the consistent retrieval of all raisins and the absence of simultaneous slowing on the Brinkman Board and impairments on CANTAB made that less likely.

NHP models are useful in the study of viral neuropathogenesis as they are closely related to humans, especially with respect to brain pathology, which is typically difficult to investigate in humans. To our knowledge, this is the first report where there is concomitant evaluation of the impact of Zika virus infection in adult rhesus macaques on soluble and cellular markers of immune activation in the CSF and assessment of neurocognition. Neurocognitive evaluation by CANTAB in NHP permits translation into humans as the instrument utilizes a cross-species cognitive test battery. This method can be applied to a diverse range of infectious as well as non-infectious conditions where there is reasonable clinicopathologic correlation between NHP models and clinical disease in humans.

## Conclusions

In summary, Zika virus infection in adult rhesus macaques was associated with transient plasma viremia and immune activation in the peripheral blood and CSF. Importantly, neurocognitive impairment was identified in the majority of animals. The co-occurrence of systemic and neuro-immune perturbations and neurocognitive impairment establishes the utility of this model to further study the impacts of neuroinflammation on neurobehavior in rhesus macaques.

## Supplementary Information

**Additional file 1: Fig. S1.** Monkey CANTAB Intellistation with Pellet Reward training procedures.

**Additional file 2: Table S1.** Pre-infection Neurocognitive Performance.

## Data Availability

The data generated or analyzed during this study are included in this published article.

## Abbreviations

ZIKV: Zika virus
CANTAB: Cambridge neuropsychological test automated battery
CSF: Cerebral spinal fluid
PI: Post-inoculation
DENV: Dengue virus
YFV: Yellow fever virus
JEV: Japanese encephalitis virus
WNV: West Nile virus
TBEV: Tick-borne encephalitis virus
NHP: Nonhuman primate
AFRIMS: Armed Forces Research Institute of Medical Sciences
MOT: Motor screening task
SOSS: Self-Ordered Spatial Search
HAI: Hemagglutination inhibition
MEM: Minimum Essential Media
RT: Room temperature
PBMC: Peripheral blood mononuclear cells
DPBS: Dulbecco’s phosphate buffered saline
SD: Standard deviation
URTIs: Upper respiratory tract illnesses
