# Supplementary figures and images for: Neurocognitive impact of Zika virus infection in adult rhesus macaques
Source: J Neuroinflammation. 2022 Feb 7;19:40. doi: 10.1186/s12974-022-02402-4 (PMC8822695; doi:10.1186/s12974-022-02402-4)

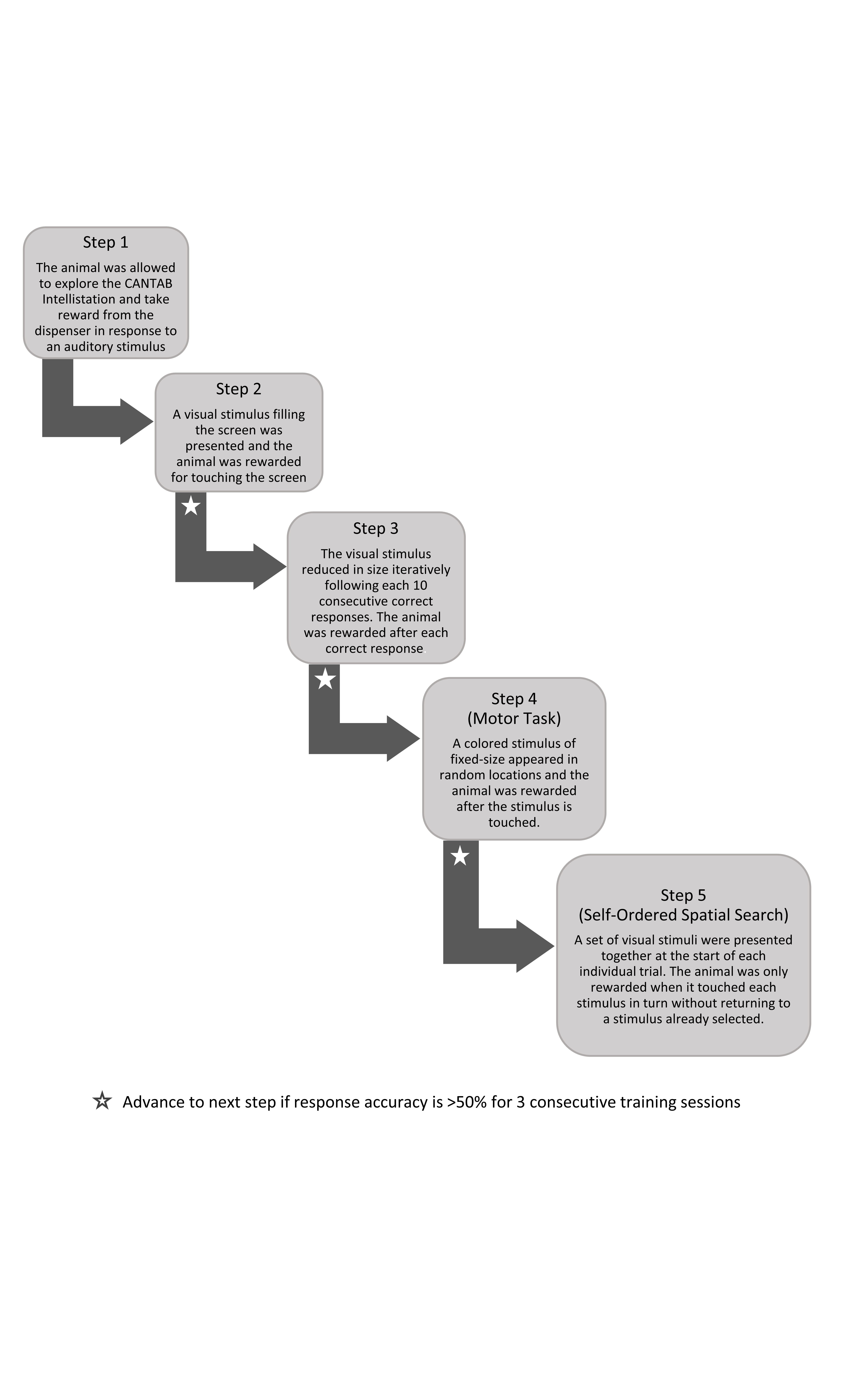

Supplement: Supplementary file 1 — Additional file 1: Fig. S1. Monkey CANTAB Intellistation with Pellet Reward training procedures. [file 12974_2022_2402_MOESM1_ESM.jpg]

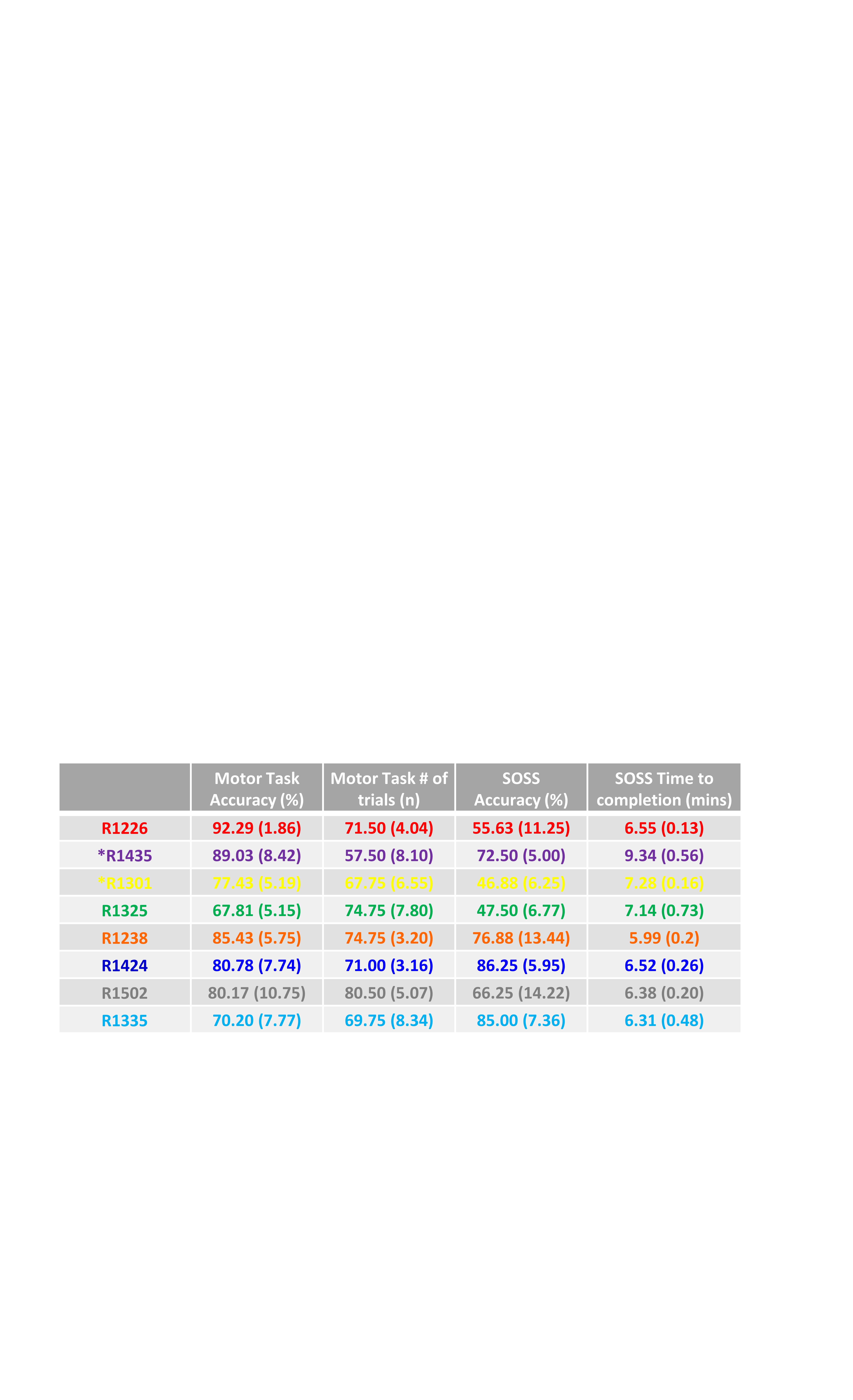

Supplement: Supplementary file 2 — Additional file 2: Table S1. Pre-infection Neurocognitive Performance. [file 12974_2022_2402_MOESM2_ESM.jpg]
